# Silicon Dioxide Thin Film Mediated Single Cell Nucleic Acid Isolation

**DOI:** 10.1371/journal.pone.0068280

**Published:** 2013-07-10

**Authors:** Evgeny Bogdanov, Irina Dominova, Natalia Shusharina, Stepan Botman, Vitaliy Kasymov, Maksim Patrushev

**Affiliations:** 1 Immanuel Kant Baltic Federal University, Kaliningrad, Russia; 2 University College London, London, United Kingdom; Indian Institute of Science, India

## Abstract

A limited amount of DNA extracted from single cells, and the development of single cell diagnostics make it necessary to create a new highly effective method for the single cells nucleic acids isolation. In this paper, we propose the DNA isolation method from biomaterials with limited DNA quantity in sample, and from samples with degradable DNA based on the use of solid-phase adsorbent silicon dioxide nanofilm deposited on the inner surface of PCR tube.

## Introduction

The problem in isolating DNA is the limited DNA quantity in sample, which is insufficient to carry out extensive analysis. DNA output from samples can be a limiting factor, especially in single cell diagnostics, forensic biology and molecular archaeology, in these cases samples contain trace amounts of DNA. Single cell diagnostic is an important field for research, and is offered use in preimplantation genetic diagnostic, forensics, oncology, and other areas [1]. For instance, high-throughput genetic analysis demands special requirements to quantity and quality isolated DNA. In particular genome-wide single-nucleotide polymorphism (SNP) assay for the detection of a disease requires up to 10 ng of DNA sufficiently per a SNP assay.

Tradition methods of single cell DNA isolation are based on cell lysis in distilled water and subjected to freeze-thawing. Since this DNA isolation method was not effective and resulted in declining the effectiveness of amplification, another technique of a two-step lysis was developed. This method of DNA extraction aimed at improving the efficiency of amplification, but it requires a lot of precise manipulations resulting in increased levels of false-negative results [2]. Also, a great number of manipulations lead to partial or complete loss of the DNA and its probable contamination. Therefore, it is necessary to develop a new nucleic acids isolation method from single cells that would involve minimal manipulation and allow for the sample transfer from one vessel to another.

Commonly used methods for isolation and purification of nucleic acids rely on the principles of solid-phase extraction, which is based on the ability of nucleic acids (NA) for adsorption on the silicon dioxide (IV) surface. The nucleic acid binding technique by silicon sorbents in high ionic strength solutions was developed in 1990. Now it is used in many commercial kits and automatic systems for the nucleic acids isolation [3], [4], [5], [6], [7], [8], [9], [10].

Thus, on the basis of the recent research outcome, we developed a new nucleic acids isolation method from a single cell using the PCR tube covered by a silicon dioxide thin film (SDTF). Functional coatings on the inner surface of PCR tubes can be used for nucleic acids adsorption. This property of SDTFs has not been studied before [11].

## Materials and Methods

### PCR tube preparing

A polyfunctional ion beam deposition (IBD) system [12] consisted mainly on one of Kaufman ion source with neutralizer and one target holder that had been used to synthesize SDTFs. In experiments, the base pressure of the chamber was below 4,5×10^−5^ Pa, the working pressure was 5×10^−2^ Pa. A commercially available 25 cm silicon dioxide disk (“Ligamet”, Russia) was used as the ion beam sputtering target. PCR tubes were exposed with the open side facing the path of sputtering target. For characterization purposes, graphite samples were also included in one process of coating run. The deposited silicon dioxide monolayer coatings were produced using an Ar^+^ beam of 1000 eV and 20 mA cm^−2^ for 50 min, 5 min, 2 min, 1 min, 30 sec. The thickness of all the coatings were about approximately 250 nm, 24 nm, 10 nm, 5 nm and 3 nm respectively. The energetic ion beam was produced by ionizing high purity argon gas (99.999% pure).

Auger-electron spectroscopy (AES) with ion profiling was employed to analyse the chemical structure deposited on inner surface PCR tube coatings. Raman scattering spectroscopy was also used to phase analysis of silicon dioxide.

For the AES analysis of silicon dioxide on PCR plastic tube samples the silver (Ag) thin film was synthesized as conductive indicator layer by IBD with using Ar^+^ beam of 1000 eV and 20 mA cm^−2^ for 30 sec.

In order to determine the distribution of elements in coatings on graphite and plastic samples with various SDTFs thickness AES depth profile was accomplished by sputter etching the coated samples with the etching step 1,2 nm/min and Ar^+^ ion energy 500 eV. Analysis of the thin films was performed until the PCR tube surface was reached. Also AES analysis was used for mapping elements of silicon dioxide covering PCR tube inner surface.

The Raman spectra of SDTF on two random PCR tubes with 250 nm covering were obtained under identical experimental conditions: 632.8 nm wavelength excitation source, 10 s integration time and 100 mm aperture size. Phase control was carried out from PCR tube inner surface.

### Ethics Statement

Isolation of oocytes from rats was carried out in strict accordance with the directive of the Ministry of Health and Social Development of the Russian Federation dated 23 August 2010 #708n “On approval of the laboratory practices”. The protocol was approved by the Committee on the Ethics of Animal Experiments of the Immanuel Kant Baltic Federal University (Order Number: 347-12).

### Isolation of oocytes from rats

Single cells (oocytes) were obtained from laboratory rats *Rattus norvegicus speciesn*. After euthanasia by CO_2_ inhalation female rats ovaries were carefully removed and placed in pre-warmed (37°C) M2 medium («Sigma-Aldrich», USA). Cumulus-oocyte complexes (COCs) were retrieved by flushing ovaries in this solution. To remove cumulus cells, the COCs were incubated in M2 medium containing 1 mg/ml bovine testicular hyaluronidase («Sigma-Aldrich», USA) at 37°C for 10 mins and further washed twice using fresh M2 medium [13], [14], [15].

### DNA isolation from a single cell using tubes with SDTF

For DNA isolation using PCR tubes with SDTF, we transferred single oocyte in tubes by micromanipulator («Eppendorf», USA). Cells were lysed by 0.05 M Tris-HCl (pH = 6.05) («Amresco», USA) with 0.25 M NaCl («Helicon», Russia) solution, subsequently the reagent mixture was heated at 95°C for 10 mins. Then the mixture was cooled and vortexed for 1 min and incubated at room temperature for 10 mins for the effective DNA adsorption, and then the liquid was discarded. Washing from impurities was carried out by the mixture of Tris-HCl (0.05 M) and NaCl (0.25 M). The solution was discarded and the tube was slightly dried.

### Real-time Polymerase chain reaction

The real-time polymerase chain reaction (RT-PCR) was carried out on CFX96 Real-Time PCR Detection System («Bio-Rad», USA) in PCR tube with SDTF, containing 20 µl of the reaction mixture: buffer for Taq DNA Polymerase («Evrogen», Russia) –1x, MgCl_2_ solution –1,8 mM, solution of dNTP –0,25 mM, primer for rat mitochondrion gene ND5 identification (Forward: 5′ –AGA AGG CCC AAC TCC CGT CTC TG–3′; Reverse: 5′ –GGC CTA GTT GGC TGG ATG TTG AG–3′, probe: Cy5-TA GCA GGG ATC TTC CTA ATAATC C-BHQ2) –0.3 mM, Taq polymerase («Syntol», Russia) –1 u. The cycler real-time CFX 96 was started under the following reaction conditions: 95°C for 3 min (once), 95°C for 10 seconds, 56,3°C for 30 seconds, 72°C for 40 seconds (50 cycles).

### Adsorbed DNA quantification

Lambda DNA was used as a control to measure the molecules adsorption. For fluorometric analysis DNA was eluted from SDTF with mixture of 0.01 M Tris-HCl and 0.002 M EDTA («Amresco», USA), and amount of DNA was measured using Qubit 2.0 System («Invitrogen», USA) in the eluate.

## Results

### Characterisation of SDTF on the inner surface of a PCR tube

**Figure 1:**
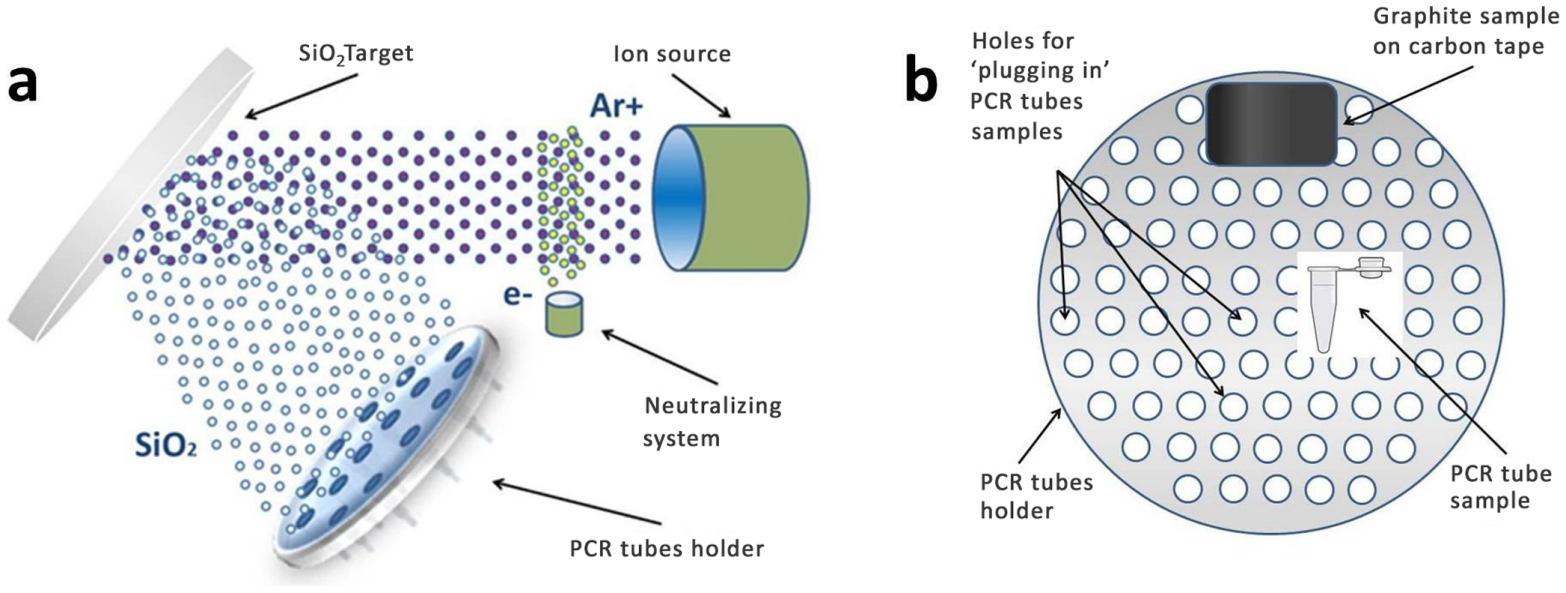
Schematic principles of A) SDTF synthesis on inner surface PCR tube by ion beam deposition method, B) PCR tubes holder and samples configuration for silicon dioxide IBD.

The IBD method allows the creation of high-quality coatings with a much higher adhesive strength to the substrate, compared to a traditional coating method. It was also the advantage of a low substrate temperature and high reliability and reproducibility, without adversely affecting the bulk attributes [16], [17], [18], [19], [20]. Schematic illustration of IBD is represented in (Figure 1A). The tubes, which were tapered at the close end, were pushed into the holes in the purpose-designed large-scale planet-type sample holder such that the top entry orifice is aligned normal to the surface (Figure 1B).

**Figure 2:**
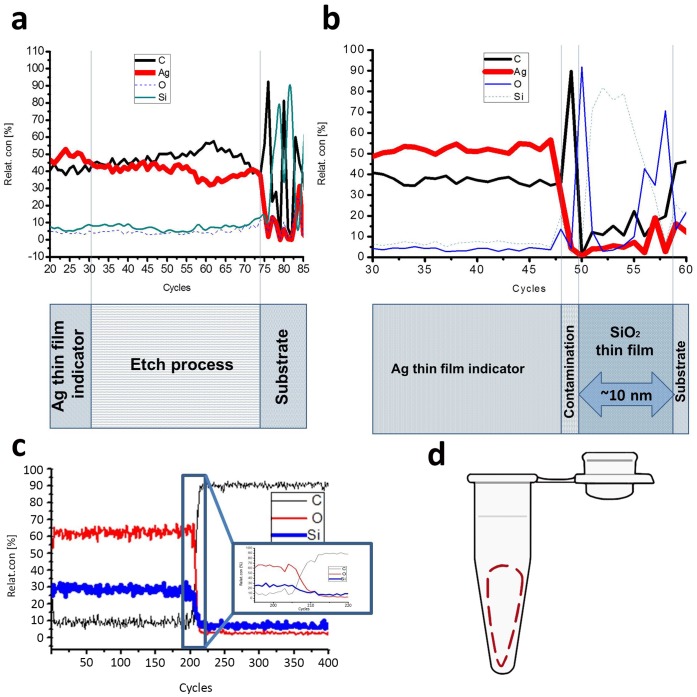
SDTF chemical structure and plastic (polypropylene) sample with functional covering scheme. A) The result of ion etching of thin films layers of Ag(indicator)/SiO_2_(silicon dioxide)/C(polypropylene)-substrate by Auger electron spectroscopy thin films ion profiling PCR tube inner surface sample. The spectrum indicates the chemical elements surface concentration by results of which is observed the absence of silicon dioxide on the PCR tube inner surface. B) Ag(indicator)/SiO_2_(silicon dioxide)/C(polypropylene)-substrate surface concentration on inner surface of PCR tube after AES Ion profiling of thin films. The spectrum indicates an increase of the silicon and oxygen peaks. C) Auger electron spectroscopy depth profile of SDTF on graphite substrate. D) The dashes line show the research area of PCR tube inner surface.

After PCR tube preparation we investigated the structural chemical and phase properties of the silicon dioxide coating. The composition profiles near the interface between coating and substrates were determined by AES. The 10 nm coating thickness for AES analysis was controlled to be about 8 min. Typical depth profiles of the samples prepared by IBD are presented in (Figure 2A, B, C). According to the depth profile data of 250 nm silicon dioxide coating on graphite substrate (Figure 2C) we can estimate relative concentration of Si:O equal 1∶2 and observe that the structure was free from impurities and mixings. This confirms the fact of silicon dioxide film presence on experimental samples.

The silicon dioxide coating thickness on PCR tube inner surface and flat graphite sample is different after one IBD process of coating run. The plastic (polypropylene) samples were cut from PCR tubes with functional coverings for silicon dioxide thickness measurement (Figure 2D). AES analysis spectra of SDTF on inner surface PCR tube presented on (Figure 2A, B).

In-depth profile of the sample with silver indicator layer on SDTF equals approximately 5 nm. After indicator layer (Ag) etch SDTF is not observed. (Figure 2A). Another AES depth profile of silver indicator thin-film layer on silicon dioxide (∼10 nm) coating shows decrease of Ag relative concentration after 47 cycles and increase of silicon oxide relative concentration after 50 cycle (Figure 2B). The contamination is due to poor cleaning of the sample prior to Ag deposition.

All tubes were tested by PCR. From experiments we conclude that PCR tubes with silicon dioxide layer thinner than 10 nm can not be used for single cell nucleic acid isolation using extraction method described above. This caused by irregular coating distribution on PCR tube inner surface.

**Figure 3:**
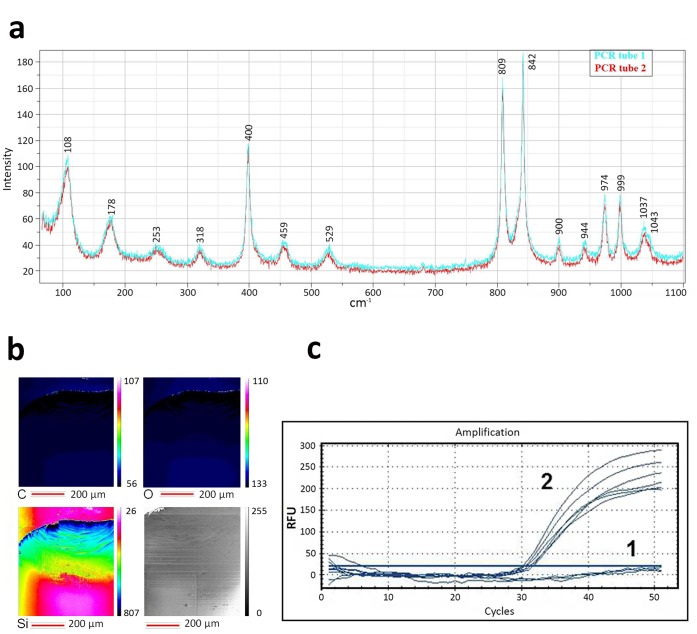
Phase analysis, element distribution mapping of plastic (polypropylene) sample with functional covering and real-time polymerase chain reaction. A) Raman spectrum of two samples of inner surface PCR tubes with amorphous SDTF prepared by the IBD method under the same conditions. B) Auger electron spectroscopy element distribution mapping. Scale bar is 200 µm. The spectrum color scheme shows distribution of: carbon (C) – upper left scan sector, oxygen (O) – upper right scan sector, silicon (Si) – lower left scan sector and secondary electron image (lower right scan sector). C) Amplification curves obtained using 1– approach, which involves the introduction of a single cell (oocyte) in a tube without silicon dioxide nanocovering; 2– method implied the introduction of a single cell (oocyte) in a tube with silicon dioxide nanocovering, followed by a cell lysis and washing of tubes from impurities. It gave uniform positive results in the course of PCR, which confirms the efficient extraction of DNA from single cells.

Raman scattering spectroscopy was used to verify that the silicon dioxide crystalline phase was absent. In (Figure 3A) exhibited only the polypropylene substrate spectra [21] of two plastic samples with functional covering. This result confirmed the fact that SDTF structures on PCR tubes are not crystalline. Raman scattering analyses showed that the SDTFs on the PCR tube inner surface synthesized by IBD were amorphous. This “amorphous” appearance is a direct result of the ion beam deposition technique. Because of the high vacuum and a room temperature, there was not enough energy for the growth of nano-crystallites in the coatings during the deposition process, and hence the silicon dioxide coatings were shown to be amorphous.

AES element mapping results obtained from the surface of SDTF 10 nm on PCR tube were allocated regularly but unevenly in terms of stoichiometry (Figure 3B). Irregular allocation is caused by the thin film coating synthesis technique features. Carbon (C) contamination caused by substrate preparation for the investigation. The film deformation on a plastic sample was caused by a piece of PCR tube being cut off (Figure 3B). The silicon dioxide regular allocation is a prerequisite for single cell nucleic acid isolation. An apparent effect on the thin films stoichiometry to the DNA extraction was not found.

We further studied the method of a single cell DNA isolation in a PCR tube with SDTF to establish the optimal conditions and give the highest output of DNA isolation. Estimate of the absorbed DNA amount on SDTF indicated that only 1.0% of the introduced DNA was absorbed on the inner surface of the PCR tube, because of the surface area of SDTF is limited by the inner tube area. Magnetic particles covered by silicon dioxide can be used to solve this problem that developed on the surface area. But at the same time, single cell DNA isolation is complicated by using this method because it has many imperfections such as: sensitivity to factors such as pH, temperature, buffer compositions, that often require dynamic control in order to minimize DNA losses. Negatively charged proteins also can decrease the efficiency of isolation by interaction with silicon dioxide surface [22]. Inability to use magnetic particles for single cell DNA isolation was confirmed by us in a number of experiments. We introduced single cells in tubes with lysis buffer (same as the isolation DNA per PCR tubes with SDTF) and added to mixture magnetic particles covered by silicon dioxide. The obtained results demonstrated that particles adsorbed much less single cell DNA than thin film. Moreover our method allows a reduction on the duration of DNA extraction and number of manipulations.

To confirm the DNA extraction real-time PCR was carried out in the PCR tube, containing adsorbed DNA molecules on its inner surface. As a control we used simple approach applying the introduction of an oocyte in a tube without SDTF.

Based on these results, we found that the control approach is not effective (Figure 3C (1)). Accordingly, this technology does not allow DNA extraction from single cells.

The extraction method gave uniform positive results in the course of PCR, which demonstrates the efficiency of DNA extraction from single cells (Figure 3C (2)). Data shown demonstrates small threshold cycles at 30–32, which was explained by small quantity of input DNA. These results were confirmed in 150 experiments with negative controls, which gave representative data.

## Discussion

We have developed a method for DNA extraction from a single cell using PCR tubes with SDTF covering. PCR verification demonstrated the suitability of this method for DNA isolation. We supposed that the mechanism of binding DNA by SDTF is the same as on silicon dioxide particles, which is showed in the research by Melzak et al. It represents three competing effects between nucleic acids and silica:

- weak electrostatic forces of repulsion;
- dehydration;
- formation of hydrogen bonds [23].

The DNA adsorption occurs on the silicon dioxide surface at the lysis temperature at 95°C. Diesters of phosphates on the DNA chain are strong acids, making double-stranded DNA strong polyelectrolyte, which carries two monovalent negative charges on the base pair in the majority of the pH values. High temperature and Tris-HCl solution affects the DNA structure, breaking hydrogen bonds molecule with the formation of single-stranded molecules. Released nitrogenous bases of single-stranded DNA can form hydrogen bonds with the SDTF. It is the driving force which compensates for the weak electrostatic repulsion between nucleic acid and the sorbent [24].

Monovalent cations (sodium ions) are bound to the DNA chain, thus the DNA negatively charged surface is neutralized through the condensation of counterions. The surface of the silicon dioxide is also negatively charged at the alkaline and near-neutral pH value due to weak acidic silanol groups. The surface silanol groups are usually titrated within a wide range of pH values from 4 to 8 with an average pKa between 5 and 7. Positive sodium ions form bridges between two negatively charged surfaces. It allows the DNA molecule to move closer to the negatively charged surface, in this case the electrostatic interactions are not strong enough, so the adsorption can occur under van der Waals forces [8], [10], [25].

Intermolecular electrostatic forces can make significant contributions to the adsorption mechanism, especially at near and above neutral pH values. The network of electrostatic repulsion between DNA fixed charges and silicon surface charges will create conditions (low ionic strength solution), in which there will be poor adsorption [24], [25].

Also, we developed the technology for DNA extraction from a single cell using PCR tube as a base. We concluded that the ion beam deposition is a very flexible method for growing silicon dioxide coatings on the inner surface of a PCR tube. In general, silicon dioxide coating formation on the inner surface of a PCR tube is not limited by ion beam deposition method. We suggest that any thin-film synthesis methods can be used for such purposes. However, PCR tubes must not be overheated and the functional covering must be evenly distributed on the inner surface of the PCR tube. For example, such methods could include pulsed laser deposition, magnetron sputtering and atomic layer deposition. Based on this, we can assume that, besides silicon dioxide some other oxide coatings (tantalum, niobium, aluminum, titanium oxides) can be used for the coating of the inner surface of PCR tubes. However, this assumption must be verified.
